# Dr. Henry R. Shine

**Published:** 1891-06

**Authors:** 


DEATH OF DR. HENRY R. SHINE.—Dr. Henry R. Shine died in Tallahassee, Fla., at nine o'clock Thursday morning, July 15, 1891, after a painful illness of many months.

He was the son of Mr. and Mrs. R. A. Shine, and was born in Tallahassee, August 25th, 1867.

As a boy he was recognized as one of the brightest at school, and at fifteen years of age was made Captain of the West Florida Cadets, and then promoted to the office of Adjutant. After he came back from College he was made Captain of the Governor's Guards, of Tallahassee. He was pronounced by competent judges to be one of the most gifted officers in the State.

At the age of seventeen he attended lectures in the Dental Department of the University of Maryland, and graduated at nineteen with distinction.

His dexterity in the use of soft foil called forth the highest compliments of the professors, demonstrators, and his fellow students. They pronounced him a genius.

Dr. Shine established a lucrative practice and was held in high esteem as a skillful dentist, and won the confidence and esteem of all who knew him. The profession has lost one of its most promising and useful members. Such men shall be sadly missed.

We did not know Dr. Shine personally, but our business relations with him were so pleasant and satisfactory we readily recognized the gentleman in every sense of the word. We deeply sympathize with his bereaved family.

The following complimentary words are copied from the Tallahassee *Press*:

'The soul of Dr. Henry R. Shine was called from its earthly abode Thursday morning, to a sphere of higher usefulness. The funeral took place from St. John's Episcopal Church, Friday afternoon, Rev. W. A. Carter officiating, attended by a large concourse of friends and relatives of the family.

"He was a graduate of the University of Maryland, Dental Department, and stood high in the profession.

"He was of a genial, sunny disposition, and was beloved by all who came in contact with him. His high moral worth and soft gentlemanly manners made mothers point him out to their sons as an example worthy of trying to follow."

Death always causes a certain degree of solemnity in a community, but in this instance, though not unexpected, the feeling of sincere regret and spontaneous sympathy is universal, for he surely possessed many traits of character which endeared him to all his acquaintances, while strengthening the ties of love, admiration and affection on the part of his devoted parents and family connections. His young life was so full of promise. He was so manly in his bearings, so unassuming in manner, so courteous and affable in deportment, so correct in business, that he had won the confidence and esteem of the entire community, and older men regarded him as one on whom his country could rely as a safe counsellor and ready defender in any emergency.

During his long and painful illness, he was most tenderly cared for by loving parents, brothers and sisters, into whose grief stricken home we would not intrude, save a tender and heartfelt condolence of a sympathetic community.—*Dental Luminary.*

---

